# Factitiously Elevated Total Triiodothyronine in a Euthyroid Patient with Multiple Myeloma

**DOI:** 10.1155/2021/8479193

**Published:** 2021-08-30

**Authors:** Kaushik Mandal, Damilola Ashorobi, Alice Lee, Huijuan Liao, Salini C. Kumar, David S. Rosenthal

**Affiliations:** Division of Endocrinology, Nassau University Medical Center, 2201 Hempstead Turnpike, East Meadow, NY 11554, USA

## Abstract

Sporadic reports of factitious elevations of thyroid hormones related to laboratory interference from autoantibodies and multiple myeloma paraproteins have appeared in the literature. Such clinically confusing laboratory results can lead to erroneous diagnoses and inappropriate treatments. We report an additional case of a patient with multiple myeloma and an IgG paraproteinemia who had such a spurious elevation of total T3 complicating her levothyroxine management of hypothyroidism. In addition, we alert clinicians that differences in performance characteristics between various manufacturers' test platforms may also cause spurious reports.

## 1. Introduction

More than 99% of both total T3 (TT3) and total T4 (TT4) are bound for transport to either thyroxine-binding globulin (TBG), transthyretin (TTR), or albumin [1]. Anomalous hormone binding, such as that, may be, caused by interfering autoantibodies including Human Anti-Mouse Antibody (HAMA), or paraproteins such as those found in coexisting Multiple Myeloma (MM) may yield factitious and clinically inconsistent reports possibly leading to inappropriate diagnoses and treatments. Susceptibility to such spurious reports may also vary between test platforms used by the clinical laboratory. We report such a case to alert clinicians to this issue.

## 2. Case Report

A 72-year-old woman presented to our medical center with an altered mental status having suffered a fall. She had a history of primary hypothyroidism for many years treated with a stable dose of levothyroxine 0.075 mg/d. She was clinically euthyroid without tremors, palpitations, tachycardia, or other findings suggesting thyrotoxicosis. A pathologic fracture of her right humerus eight years before had led to a diagnosis of multiple myeloma with an IgG kappa M spike paraprotein. She received chemotherapy and went into remission. However, a recent reexacerbation had occurred, and she was being considered for further therapy. Routine admission labs revealed hematocrit 20.9% (normal 38–47%), sodium 132 mmol/L (136–145), potassium 4.0 mmol/L (3.5–5.1), chloride 104 mmol/L (98–107), urea nitrogen 45 mmol/L (20–31), and creatinine 2.8 mg/dL (0.6–1.0). Oncologic studies were consistent with multiple myeloma with an M spike kappa IgG paraprotein: total protein 10.1 g/dL (5.7–8.2), albumin 1.8 g/dL (3.4–5.0), and serum albumin: globulin ratio: 0.22 (0.60–1.50). Serum protein electrophoresis revealed gamma globulin 5.8 g/dL (0.8–1.7), kappa chain 9270 mg/L (3.3–19.4), Lambda chain 9.3 mg/dL (5.7–26.3), K/L ratio 996.7 (0.26–1.65), and B2—macroglobulin: 17.9 mg/L (<or = 2.51).

Thyroid biochemistry (Table 1) on presentation performed with the Siemens Atellica chemiluminescent immunoassay kit reported a slightly high TSH of 6.27 uIU/mL (normal 0.55–4.78) with normal total T4 of 4.5 *μ*g/dL (3.2–12.6) and free T4 1.22 ng/dL (0.89–1.76). However, total T3 was markedly elevated at >600 ng/dL (60–181). Tests for anti-ThyroPerOxidase (AntiTPO), anti-ThyroGlobulin (antiTG), Thyroid-Stimulating Immunoglobulin (TSI), and Human Anti-Mouse Antibodies (HAMA) were within normal limits at <1 IU/mL (<9), <1 IU/mL (<1), <89% (<140), and <56 ng/mL (<74), respectively. Assay for anti-T3 antibodies was negative using a proprietary chemiluminescent competitive immunoassay procedure performed by Quest/Nichols Laboratories. Repeat testing on the Siemens Atellica platform again reported an elevated TT3 >600 ng/dL. Reverse T3 (Thermo Aria platform) was normal at 21 ng/dL (8–25), while results for TSH, total T4, and free T4 were similar to those reported previously. Free T3 was low at 1.7 (2.3–4.2 pg/ml (Siemens Advia kit) consistent with Euthyroid Sick Syndrome. 14 months earlier, her thyroid biochemistry was reported to be normal using the Roche Cobas chemiluminescent immunoassay kit with total T3 82 ng/dL (80–200). Thyroid Hormone-Binding Protein (THBP) electrophoresis was normal: TBG 14.3 mcg T4/dL (10.3–24.9), albumin 16.6 mcg T4/dL (11.5–34.1), and transthyretin (prealbumin) 69 mg T4/dL (48.8–70.4). Follow-up testing on the Roche Cobas platform again reported normal total T3 47.4 ng/dL (Table 1). Polyethylene glycol (PEG) precipitation was planned, but the patient expired before it could be accomplished.

We conclude that the factitious elevation in TT3 was most likely due to assay interference from the documented multiple myeloma paraprotein as well as differences between the test platforms used.

## 3. Discussion

We report a case of a clinically euthyroid patient with longstanding hypothyroidism treated with a stable dose of levothyroxine who was reported to have a factitiously elevated TT3 during an exacerbation of multiple myeloma with an IgG kappa M spike paraprotein having been identified. More than 99% of both TT3 and TT4 are bound to either thyroxine-binding globulin (TBG), transthyretin (TTR), or albumin [1]. In our case, Thyroid Hormone-Binding Protein (THBP) electrophoresis was normal. Test interference from Human Mouse Antibodies (HAMAs), anti-T3 antibodies, or antibodies to TPO or TG was ruled out. Thyroid biochemistry 14 months earlier using the Roche Cobas platform had reported a normal TT3, while both initial and follow-up tests at our institution using the Siemens Atellica kit reported spuriously elevated TT3 values. Once again, repeat analysis on the Roche Cobas system documented a normal TT3 level. Varying susceptibility to such spurious results between different manufacturers' platforms has been previously reported [2] and may in part be related to differences in antigen capture characteristics between the monoclonal antibodies used by each test kit manufacturer involving either TT3, TT4, or both.

Interfering substances such as autoantibodies and paraproteins can be removed by polyethylene glycol (PEG) precipitation followed by reanalysis. Sadly, our patient expired from her multiple myeloma before that could be carried out. We conclude that the most likely scenario in this case is interference in the assay caused by the patient's myeloma IgG paraprotein immunoglobulin identified on serum protein electrophoresis. Such immunoglobulins can bind with high affinity to T3 and/or T4 capture antibodies used in the laboratory assay which may result in falsely high values for T3 (as in this case) or T4.

Cissewski et al. [3] first described a case of factitious hyperthyroxinemia in multiple myeloma. IgA monoclonal antibody was bound to both T4 and T3 producing euthyroid hyperthyroxinemia with elevations of both TT3 and TT4.

Antonopoulou and Silverberg [4] reported a case of spurious T3 thyrotoxicosis unmasking multiple myeloma. In this report, a 54-year-old clinically euthyroid woman with normal TSH and TT4 was evaluated for isolated elevation of TT3 >800 ng/dL (60–181). Laboratory studies confirmed the diagnosis of multiple myeloma, and the authors ascribed the abnormal immunoglobulin as the cause of the assay interference yielding a spurious result.

Ram et al. [5] reported another case of factitious isolated TT3 elevation. Here, a 56-year-old man was investigated for a TT3 reported to be extremely high at 800 ng/dL (39–181) while TT4 and free T3 (FT3) were normal. Thyroid function tests had been performed on the Siemens Advia Centaur chemiluminescent analyzer. Repeat assay using the Abbot Architect immunoassay also reported very high TT3 at 519 ng/d. He was ultimately diagnosed with multiple myeloma. Polyethylene glycol (PEG) precipitation of the sample yielded a normal value for TT3 of 53.25 ng/dL on the Siemens platform. The interference was considered to be due to the paraprotein immunoglobulin.

Veechika and Chitra [6] described a 52-year-old male with an isolated abnormal TT3: >456 ng/dL (62–154) (Beckman Access one-step immunoassay). He was also subsequently diagnosed with an IgG kappa monoclonal gammopathy due to multiple myeloma. The interference was resolved by analyzing the sample using the Abbott Architect two-step immunoassay.

Recently, Pan et al. [7] analyzed thyroid function tests using the Siemens Advia Centaur platform in 105 clinically euthyroid patients newly diagnosed with multiple myeloma. They found that 13 (12.38%) had isolated strikingly elevated TT3 levels >8 ng/mL (0.6–1.81). 12 had paraprotein IgG immunoglobulins, and 1 had a light-chain kappa protein. Assay results normalized upon PEG precipitation and stayed so after myeloma chemotherapy. The abnormal paraprotein immunoglobulins were felt to be the cause of the spurious TT3 reports.

Favresse et al. [8] have reviewed pitfalls in the laboratory automated immunoanalysis of Thyroid Function Tests (TFTs) and pointed out that paraproteins may have antibody-like activity binding to analytes or reagents and, thus, may behave as heterophilic antibodies. Varying susceptibility to these types of interference between different manufacturers' platforms was also emphasized. This was evident in our case using the Siemens Atellica platform but not on the Roche Cobas platform. Both are chemiluminescent competitive immunoassays and use biotinylated T3 as a reagent. However, Siemens uses anti-mouse antibodies while Roche uses anti-sheep antibodies. Our patient was HAMA negative, suggesting other interference on the Siemens platform most likely due to the presence of multiple myeloma IgG paraprotein. When interference is suspected as in this case with the Siemens platform, a polyethylene glycol (PEG) precipitation study may help to clarify.

## 4. Conclusions

The cases reported to date have shown factitious elevation of either or both TT4 and TT3 caused by multiple myeloma paraproteins. In our case, the factitious elevation was limited to TT3 in the presence of an IgG kappa M spike paraprotein. Such spurious reports of TT3 or TT4 may be the initial presentation to the clinician and can lead to misdiagnosis and inappropriate management. Awareness of this issue is essential. Therefore, when clinically inconsistent results are encountered, one should consider the presence of such paraproteins and consider PEG precipitation and repeat testing with another test platform.

## Figures and Tables

**Table 1:** Summary of laboratory results.

TFTs	Roche Cobas kit, 08/28/19	Siemens Atellica kit, 10/25/20	Siemens Atellica kit, 10/26/20	Roche Cobas kit 11/17/20
TSH	0.750 (0.27–4.2 uIU/mL)	6.27 (0.55–4.78 uIU/mL)	4.84 (0.55–4.78 uIU/mL)	1.82 (0.27–4.2 IU/mL)
FT4		1.22 (0.89–1.76 ng/dL)	1.28 (0.89–1.76 ng/dL)	
TT4	7.6 (4.5–12.0 *μ*g/dL)	4.5 (3.2–12.6 *μ*g/dL)	4.7 (3.2–12.6 *μ*g/dL)	
TT3	82 (80–200 ng/dL)	>600 (60–181 ng/dL)	>600 (60–181 ng/dL)	47.4 (80–200 ng/dL)
HAMA			<56 (<74 ng/mL)	
TPO ab			<1 (<9 IU/mL)	
TG ab			<1 (<1 IU/mL)	
TSI			<89 (<140%)	
T3 antibody			Negative (Quest/Nichols proprietary test)	
Free T3			1.7 (2.3–4.2 pg/mL) (Siemens Advia kit)	
Reverse T3			21 (8–25 ng/dL) (Thermo Aria kit)	

## Data Availability

The data used to support the findings of this study are included within the article and the cited references.

## Abbreviations

TT3: Total T3
TT4: Total T4
TBG: Thyroxine-binding globulin
TTR: Transthyretin
HAMA: Human anti-mouse antibody
MM: Multiple myeloma
AntiTPO: Antithyroperoxidase
AntiTG: Antithyroglobulin
TSI: Thyroid-stimulating immunoglobulin
THBP: Thyroid hormone-binding protein.
